# Comprehensive analyses of one-carbon metabolism related genes and their association with prognosis, tumor microenvironment, chemotherapy resistance and immunotherapy in lung adenocarcinoma

**DOI:** 10.3389/fmolb.2022.1034208

**Published:** 2022-11-11

**Authors:** Ning Zhou, Quanying Tang, Haochuan Yu, Tong Li, Fan Ren, Lingling Zu, Gang Chen, Jun Chen, Song Xu

**Affiliations:** ^1^ Department of Lung Cancer Surgery, Tianjin Medical University General Hospital, Tianjin, China; ^2^ Tianjin Key Laboratory of Lung Cancer Metastasis and Tumor Microenvironment, Lung Cancer Institute, Tianjin Medical University General Hospital, Tianjin, China

**Keywords:** one-carbon metabolism, immune cell infiltrate, chemotherapy resistance, immunotherapy, lung adenocarcinoma

## Abstract

**Background:** Lung adenocarcinoma (LUAD) is the most common type of lung cancer and is a global public health concern. One-carbon (1C) metabolism plays a crucial role in the occurrence and development of multiple cancer types. However, there are limited studies investigating 1C metabolism in LUAD. This study aims to evaluate the prognostic value of 1C metabolism-related genes in LUAD and to explore the potential correlation of these genes with gene methylation, the tumor microenvironment, and immunotherapy.

**Methods:** We identified 26 1C metabolism-related genes and performed a Kaplan-Meier and Cox regression analysis to evaluate the prognostic value of these genes. Consensus clustering was further performed to determine the 1C metabolism-related gene patterns in LUAD. The clinical and molecular characteristics of subgroups were investigated based on consensus clustering. CIBERSORT and ssGSEA algorithms were used to calculate the relative infiltration levels of multiple immune cell subsets. The relationship between 1C metabolism-related genes and drug sensitivity to immunotherapy was evaluated using the CellMiner database and IMvigor210 cohort, respectively.

**Results:** The expression levels of 23 1C metabolism-related genes were significantly different between LUAD tumor tissues and normal tissues. Seventeen of these genes were related to prognosis. Two clusters (cluster 1 and cluster 2) were identified among 497 LUAD samples based on the expression of 7 prognosis-related genes. Distinct expression patterns were observed between the two clusters. Compared to cluster 2, cluster 1 was characterized by inferior overall survival (OS) (median OS = 41 vs. 60 months, *p* = 0.00031), increased tumor mutation burden (15.8 vs. 7.5 mut/Mb, *p* < 0.001), high expression of PD-1 (*p* < 0.001) and PD-L1 (*p* < 0.001), as well as enhanced immune infiltration. 1C metabolism-related genes were positively correlated with the expression of methylation enzymes, and a lower methylation level was observed in cluster 1 (*p* = 0.0062). Patients in cluster 1 were resistant to chemotherapy drugs including pemetrexed, gemcitabine, paclitaxel, etoposide, oxaliplatin, and carboplatin. The specific expression pattern of 1C metabolism-related genes was correlated with a better OS in patients treated with immunotherapy (median OS: 11.2 vs. 7.8 months, *p* = 0.0034).

**Conclusion:** This study highlights that 1C metabolism is correlated with the prognosis of LUAD patients and immunotherapy efficacy. Our findings provide novel insights into the role of 1C metabolism in the occurrence, development, and treatment of LUAD, and can assist in guiding immunotherapy for LUAD patients.

## Introduction

Lung cancer remains one of the most prevalent cancer types and the most lethal cancer type worldwide (Siegel et al., 2022). Lung cancer is divided into two main forms: non-small cell lung cancer (NSCLC) and small cell lung cancer (SCLC). NSCLC is the most common type of lung cancer and accounts for 85% of all cases (Duma et al., 2019; Wang et al., 2019). Lung adenocarcinoma (LUAD) is the major histopathological subtype of NSCLC and accounts for approximately half of all lung cancer-related deaths (Travis et al., 2015; Behrend et al., 2021). Although several treatments have been confirmed effective in recent years, platinum-based chemotherapy, such as pemetrexed, remains the principal therapeutic for NSCLC (Schiller et al., 2002; Tan et al., 2016).

One-carbon (1C) metabolism, also known as folate metabolism, is involved in multiple physiological processes, such as biosynthesis, amino acid homeostasis, epigenetic maintenance, and redox defense (Ducker and Rabinowitz, 2017). It has been identified that 1C metabolic enzymes are upregulated in numerous cancer types (Mehrmohamadi et al., 2014). *MTHFD2* expression is associated with poor prognosis in hepatocellular carcinoma and colorectal cancer (Liu et al., 2016; Ju et al., 2019). *SHMT2* has also been identified to play a role in colorectal and lung cancer progression (DeNicola et al., 2015; Liu et al., 2021). In addition, *TYMS* is overexpressed in several cancers and is closely associated with a poor prognosis (Sasaki et al., 2013; Fu et al., 2019; Agulló-Ortuño et al., 2020; Song S. et al., 2021). Because of the essential role of 1C metabolism in cancer, inhibition of folate metabolism is regarded as an important therapeutic strategy in cancer. Several drugs targeting 1C metabolic enzymes have been successfully developed, such as methotrexate and pemetrexed (Ducker and Rabinowitz, 2017).

It has been shown that 1C metabolism can affect the function of immune cells, especially the activation of T cell (Ducker and Rabinowitz, 2017). Immune cells play an important role in the tumor microenvironment (TME). The TME includes diverse cell types, including cancer cells, noncancerous cells, as well as many other cellular and noncellular components (Duan et al., 2020). The immune and non-immune cells within the TME have been observed to regulate the proliferation, differentiation, and death of tumor cells (Mu and Najafi, 2021). In recent years, numerous studies have shown the effectiveness of targeting components within the TME alone or in combination with other therapies, including chemotherapy, radiotherapy, and immunotherapy (Hirata and Sahai, 2017; Ozpiskin et al., 2019; Liu et al., 2020).

1C metabolism can support methylation reactions by generating 1C units (also known as methyl groups). DNA and RNA methylation has been widely considered to be the best-characterized epigenetic modifications, and play an important role in the occurrence and development of tumors. DNA methylation occurs at the 5-position of cytosine (5 mC), and transcriptionally regulates the expression of target genes (Robertson and Jones, 2000). RNA methylation mainly includes three types: N6-methyladenosine (m6A), 5-methylcytosine (m5C), and N1-methyladenosine (m1A) (Song P. et al., 2021). Methylation is a reversible modification that is regulated by special enzymes, including methyltransferase (writer), demethylase (eraser), and methylation-dependent binding protein (reader) (Dai et al., 2021).

Although 1C metabolism has been shown to have important functions in the process of methylation and the resistance to pemetrexed, its role in the occurrence, development, and treatment of LUAD remains unclear. In the present study, we found that 1C metabolism is associated with the prognosis of LUAD and the effect of immunotherapy. 1C metabolism-related genes are potential biomarkers of prognosis of LUAD and can help to guide immunotherapy in LUAD patients.

## Materials and methods

### Dataset source

RNA-seq profiles (Counts and FPKM format), somatic mutation, DNA methylation, and phenotype data from The Cancer Genome Atlas (TCGA) LUAD cohort were downloaded from the UCSC Xena database (https://xenabrowser.net/datapages/) (Goldman et al., 2020). Six GSE datasets were downloaded from GEO database (https://www.ncbi.nlm.nih.gov/geo/), including GSE3141, GSE29013, GSE31219, GSE31210, GSE37745, and GSE50081. After normalizing the datasets and removing batch effects, the expression profile data was used for the subsequent analysis.

### One-carbon metabolism-associated gene collection

Based on the findings of previous studies, 26 1C metabolism-associated genes were identified (Sasaki et al., 2013; Mehrmohamadi et al., 2014; DeNicola et al., 2015; Liu et al., 2016; Ducker and Rabinowitz, 2017; Fu et al., 2019; Ju et al., 2019; Agulló-Ortuño et al., 2020; Liu et al., 2021; Song S. et al., 2021). These genes were used for further analysis, including *PHGDH, PSAT1, PSPH, FTCD, SHMT1, SHMT2, MTHFD2L, MTHFD2, MTHFD1L, MTHFD1, GCAT, SARDH, DMGDH, GNMT, BHMT, ALDH7A1, CHDH, TYMS, MTR, MTHFR, GART, ATIC, ALDH1L1, ALDH1L2, DHFR,* and *MTFMT*.

### Gene expression and prognostic analysis

The expression level differences of 1C metabolism-associated genes between 509 LUAD samples and 58 adjacent normal tissues were tested using a Student’s *t*‐test. A Kaplan-Meier analysis based on the optimal cutoff point was performed using R packages (“survival” and “survminer”) to evaluate the clinical relevance of 1C metabolism-associated genes. A univariate Cox proportional hazard regression analysis was performed to identify the risk factors among these genes. Genes with *p* < 0.05 in the Kaplan-Meier analysis or univariate Cox proportional hazard regression analysis were considered prognosis‐related genes.

### One-carbon metabolism-associated gene-based clustering and least absolute shrinkage and selection operator regression

According to the results of the Kaplan-Meier analysis and univariate Cox proportional hazard regression analysis, 7 prognostic genes in univariate analysis were actually selected based on *p*-value and hazard ratios, including *TYMS, DHFR, MTHFD1L, MTHFD1, ATIC, GNMT,* and *CHDH*. K-means consensus clustering with these 7 genes was performed to identify subgroups in TCGA cohort. Consensus clustering was employed using the R package “ConsensusCluster” (Yu et al., 2012). The details of this process were set as follows: the number of repetitions = 1,000 bootstraps; resample rate = 0.8. LUAD patients were gathered into cluster 1 (*n* = 248) and cluster 2 (*n* = 249). Similarly, consensus clustering was also performed in GEO cohort, and patients were divided into two clusters, including cluster 1 (*n* = 397) and cluster 2 (*n* = 437). Kaplan-Meier analysis was used to assess OS differences between the two subgroups in TCGA cohort and GEO cohort, respectively.

The least absolute shrinkage and selection operator (LASSO) regression was performed to identify the prognostic genes of 1C metabolism. According to the result of LASSO regression, 7 prognostic genes were finally selected, including *TYMS, DHFR, MTHFD2L, MTHFD1, ATIC, GNMT, and CHDH*. The risk score of each patient was calculated through the equation: risk score = sum of coefficients × expression level of prognostic genes. The LUAD patients were identified as two subgroups based on the median risk score, including high-risk group and low-risk group.

### Gene set enrichment analyses

To determine the different biological processes between the two subtypes, a gene set enrichment analyses (GSEA) was conducted in the Hallmark gene set “c5.all.v7.0.entrez.gmt” of MSigDB using the R package “ClusterProfiler” (Yu et al., 2012). The parameters were set as follows: number of permutations = 1,000 and *p*-value cutoff = 0.05.

### Immune infiltrate analysis

The infiltration level of immune cells was calculated through cell type identification by estimating relative subsets of RNA transcripts (CIBERSORT) and single-sample gene set enrichment analysis (ssGSEA) (Hänzelmann et al., 2013; Newman et al., 2015). CIBERSORT (http://cibersort.stanford.edu/) was used to assess the abundances of 22 immune cell types based on the RNA-seq profile of LUAD. The relative abundance of 28 distinct leukocyte subsets was also calculated through ssGSEA using the R package “GSVA”. In addition, the immunoscore of each patient was also calculated through the R package “estimate”.

### Somatic mutations and DNA methylation analysis

To assess somatic mutations between the different subtypes, the somatic mutation profile of LUAD patients was downloaded from the UCSC Xena Database (Goldman et al., 2020). The somatic mutation data were further analyzed with the “maftools” R package. Similarly, the DNA methylation profiles were also downloaded from the UCSC Xena Database and analyzed with the “limma” R package to identify differential methylation sites (Goldman et al., 2020).

### Drug sensitivity analysis

Drug sensitivity analysis was performed using the CellMiner Database (Reinhold et al., 2012). The RNA-seq and compound activity data from the DTP NCI-60 dataset was downloaded from the CellMiner Database (https://discover.nci.nih.gov/cellminer/home.do) and was further analyzed with R Software (version 4.1.2). The correlation between 1C metabolism-associated genes and drug sensitivity was calculated. The following selection criteria were used: Food and Drug Administration approval of the therapeutic or inclusion of the therapeutic in clinical trials, and *p* < 0.05.

### One-carbon metabolism-associated gene-based immunotherapy response prediction

To validate the value of 1C metabolism-associated genes in immunotherapy prediction, the IMvigor210 cohort was used to investigate the relationships between 1C metabolism-associated genes and immunotherapy response (Mariathasan et al., 2018). Data from 348 patients who were diagnosed with urothelial cancer and treated with atezolizumab were downloaded from the IMvigor210 cohort.

### Statistical analysis

Statistical tests were carried out with R (version 4.1.2), SPSS 22.0 (IBM, NY, United States), and GraphPad Prism 9.0. For quantitative data, statistical significance for normally- and nonnormally-distributed variables were estimated using a Student’s *t*-test and Wilcoxon rank-sum test, respectively. Two-sided Fisher’s exact tests were performed to analyze contingency tables. Survival analyses were performed using the Kaplan-Meier method, and the log-rank test was used to evaluate the difference between groups. A correlation analysis was performed using a Pearson correlation test. Multivariate analyses were conducted using a Cox regression model to identify the independent risk factors. A *p*-value < 0.05 was considered statistically significant.

## Results

### Expression of one-carbon metabolism-associated genes

**FIGURE 1:**
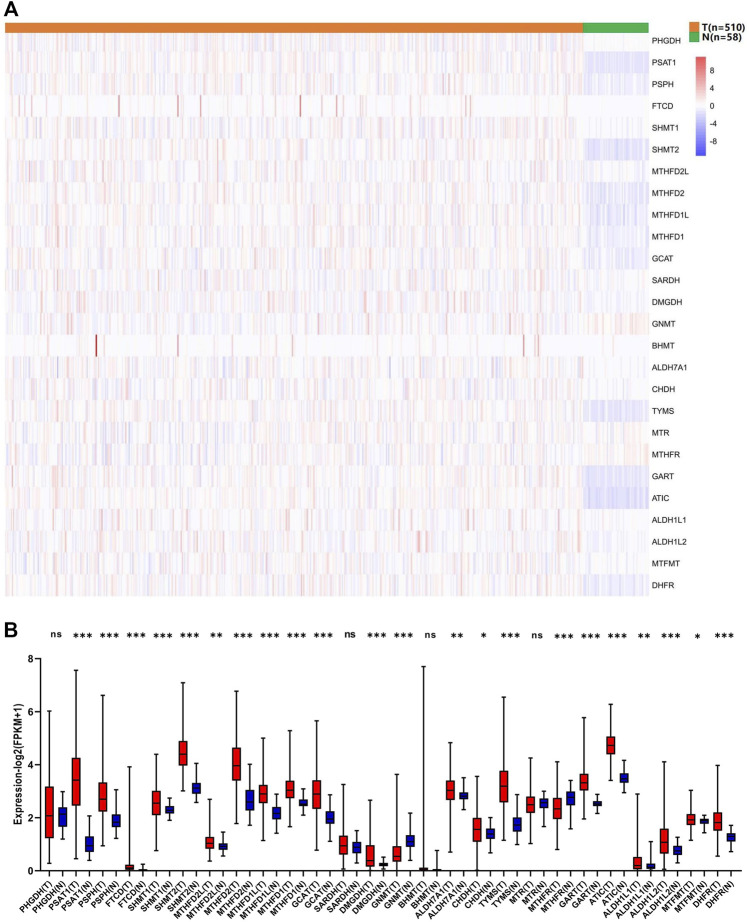
Expression levels of one-carbon metabolism associated genes in normal and tumor samples. **(A)** Heatmap of one-carbon metabolism associated genes expression level in each sample; **(B)** The expression difference of one-carbon metabolism associated genes between tumor and normal samples. * means *p* < 0.05; ** means *p* < 0.01; *** means *p* < 0.001; ns means no significant difference.

To evaluate the biological function of 1C metabolism-associated genes in the occurrence and development of LUAD, the expression pattern of 26 1C metabolism-associated genes was assessed in LUAD and adjacent normal tissues. Significant differences were observed in the expression levels of 23 genes between LUAD and adjacent normal tissues (Figure 1A). The expression level of 20 genes was upregulated, including *PSAT1, PSPH, FTCD, SHMT1, SHMT2, MTHFD2L, MTHFD2, MTHFD1L, MTHFD1, GCAT, DMGDH, ALDH7A1, CHDH, TYMS, GART, ATIC, ALDH1L1, ALDH1L2, DHFR* and *MTFMT* (Figure 1B). *GNMT* and *MTHFR* were significantly reduced in LUAD compared to adjacent normal tissues (Figure 1B). These results suggest that 1C metabolism-associated genes have important biological roles in LUAD development.

### Prognostic value of one-carbon metabolism-associated genes

**FIGURE 2:**
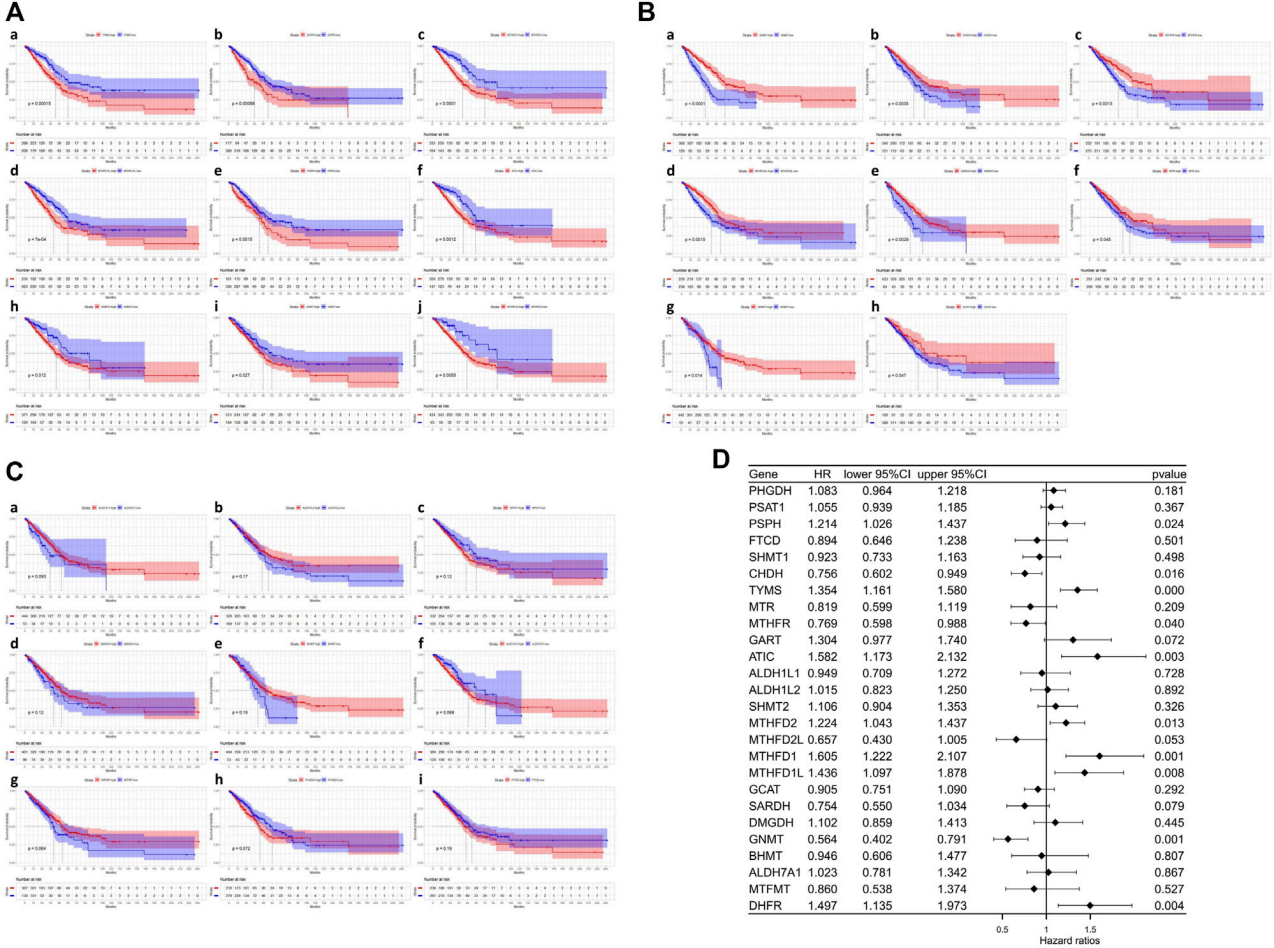
Kaplan-Meier survival curves and Univariate Cox regression analysis of one-carbon metabolism associated genes. **(A)** Kaplan-Meier survival curves of nine genes associated with inferior OS; **(B)** Kaplan-Meier survival curves of eight genes associated with superior OS; **(C)** Kaplan-Meier survival curves of nine genes not associated with OS; **(D)** Univariate Cox regression analysis of 26 one-carbon metabolism associated genes.

We further investigated the prognostic significance of 1C metabolism-associated genes in patients of LUAD in TCGA cohort. The Kaplan-Meier analysis based on an optimal cutoff shows that 17 genes were associated with OS, while nine genes were unrelated to prognosis (Figures 2A–C). The nine genes were identified as risk factors and included *ATIC, GART, MTHFD1, MTHFD1L, MTHFD2, PSPH, SHMT2, DHFR,* and *TYMS* (Figure 2A). Several other genes were considered protective factors, such as *CHDH, GCAT, GNMT, MTHFD2L, MTHFR, MTR, SARDH,* and *SHMT1* (Figure 2B). A univariate Cox regression analysis was also performed and 10 genes had a significant prognostic correlation with OS. *MTHFD2, MTHFD1L, MTHFD1, TYMS, DHFR,* and *ATIC* were risk factors, while *SARDH, CHDH, GNMT* and *MTHFR* were protective factors (Figure 2D). In GEO cohort, the Kaplan-Meier analysis which was based on the optimal cut-off revealed that 23 genes were related to OS, including 13 risk factors and 10 protective factors (Supplementary Figures S1A–C). Univariate Cox regression analysis was also performed and 11 genes were observed associated with OS (Supplementary Figure S1D). In addition, ROC curves were drawn to assess the specificity and sensitivity of 1C metabolism related-genes. The results indicated that the value at 1-, 5- and 10-year were 0.63, 0.67 and 0.74 in TCGA cohort, respectively (Supplementary Figure S2A). The AUC value of 1-year, 5-year and 10-year in GEO cohort were 0.64, 0.65 and 0.63 respectively (Supplementary Figure S2B).

### One-carbon metabolism-associated gene-based consensus clustering

**FIGURE 3:**
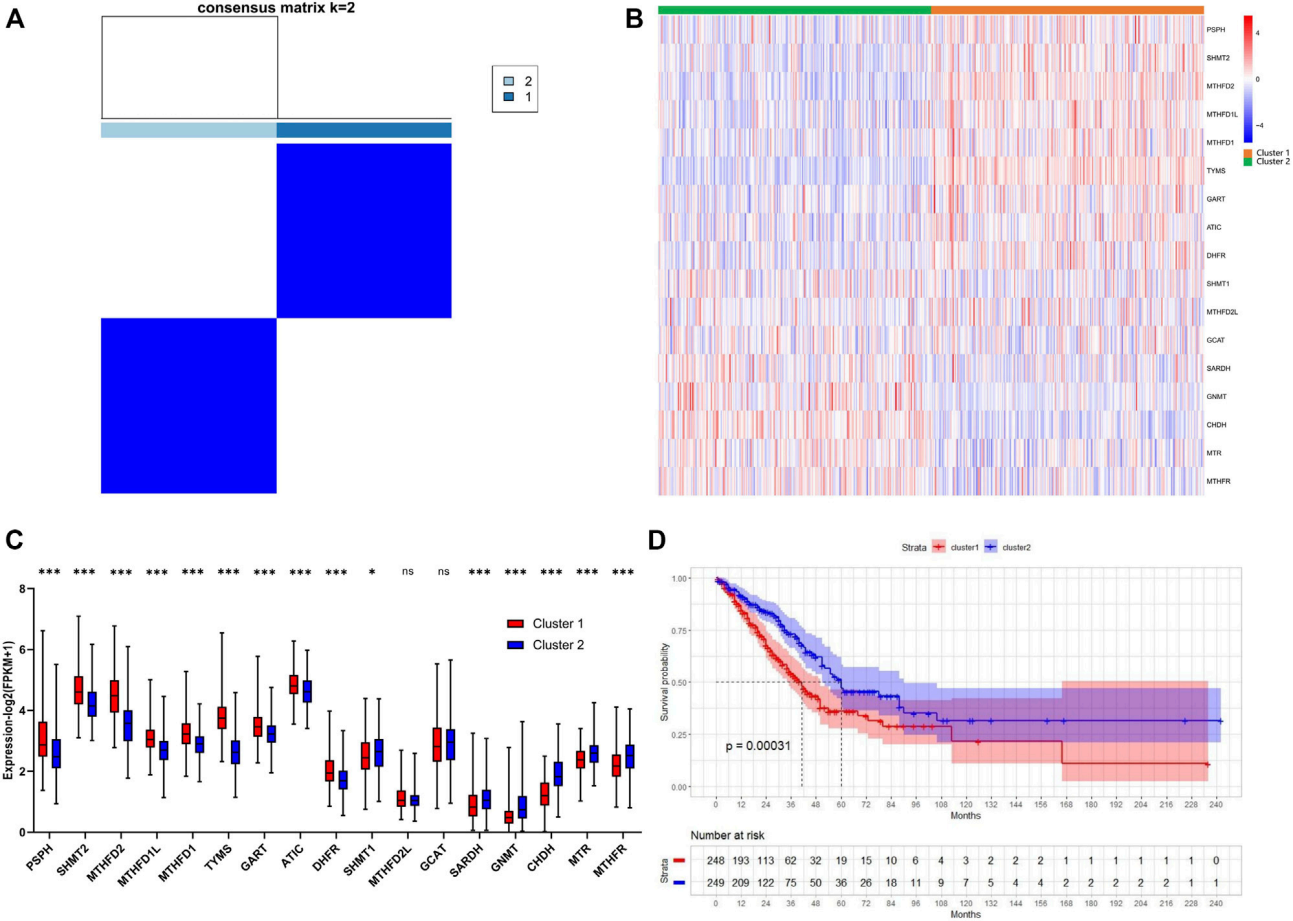
Consensus clustering for one-carbon metabolism associated genes in LUAD patients. **(A)** The consensus matrix shows patients with two distinct one-carbon metabolism statuses; **(B)** Heatmap of one-carbon metabolism associated genes expression level in two clusters; **(C)** The expression difference of one-carbon metabolism associated genes in two clusters; **(D)** Kaplan-Meier curves for overall survival in two clusters (Log-rank test). * means *p* < 0.05; ** means *p* < 0.01; *** means *p* < 0.001; ns means no significant difference.

Consensus clustering was performed to investigate the heterogeneity of 1C metabolism-associated gene in TCGA cohort. A total of 497 patients with LUAD were clustered into two subtypes. Cluster 1 (*n* = 248) was characterized by a high expression of high-risk genes while cluster 2 (*n* = 249) was identified by a high expression level of protective genes (Figure 3A). These two clusters exhibited the opposite expression pattern. Cluster 1 was characterized by high expression of *PHSH, SHMT2, MTHFD2, MTHFD1L, MTHFD1, TYMS, GART, ATIC,* and *DHFR*, as well as low expression of *SHMT1, SARDH, GNMT, CHDH, MTR* and *MTHFR* (Figures 3B,C). A Kaplan-Meier analysis showed that patients who were divided into the cluster 1 subgroup suffered inferior OS (median OS: 41 vs. 60 months, *p* = 0.0003; Figure 3D). Clinical characters between the two clusters were also investigated. Tumor metastasis (*p* = 0.016), advanced stage (*p* = 0.036), and smoking status (*p* < 0.001) were more frequently observed in cluster 1 (Table 1). Similar results were also observed according to LASSO regression and risk score model (Supplementary Figure S3A–H). In addition, consensus clustering was also performed in GEO dataset, and two clusters were identified, including cluster 1 (*n* = 397) and cluster 2 (*n* = 437) (Supplementary Figure S4A). Compared with TCGA cohort, similar expression patterns in two clusters were observed in GEO dataset (Supplementary Figures S4B,C). Kaplan-Meier analysis also revealed that cluster 1 subgroup exhibited an inferior OS in GEO cohort (median OS: 69 vs. 132 months, *p* < 0.0001; Supplementary Figure S4D).

**TABLE 1:** Clinicopathological characteristics of subgroups.

	Cluster 2	Cluster 1	P
Age			0.384
≥70 year	85	75	
	
<70 year	160	167	
Gender			0.262
Male	108	120	
Female	141	128	
Race			0.590
White	192	192	
Black	27	24	
Other	30	32	
Stage			0.036
I	146	121	
II	55	63	
III	36	44	
IV	7	18	
T stage			0.070
T1	95	71	
T2	119	148	
T3	23	20	
T4	10	8	
N stage			0.079
N0	169	152	
N1–N3	73	92	
M stage			0.016
M0	167	164	
M1	6	18	
Smoking status			<0.001
Non-smoker	47	24	
Current smoker	44	74	
Reformed smoker	152	142	

### Consensus clustering-based genetic landscape and gene set enrichment analyses

**FIGURE 4:**
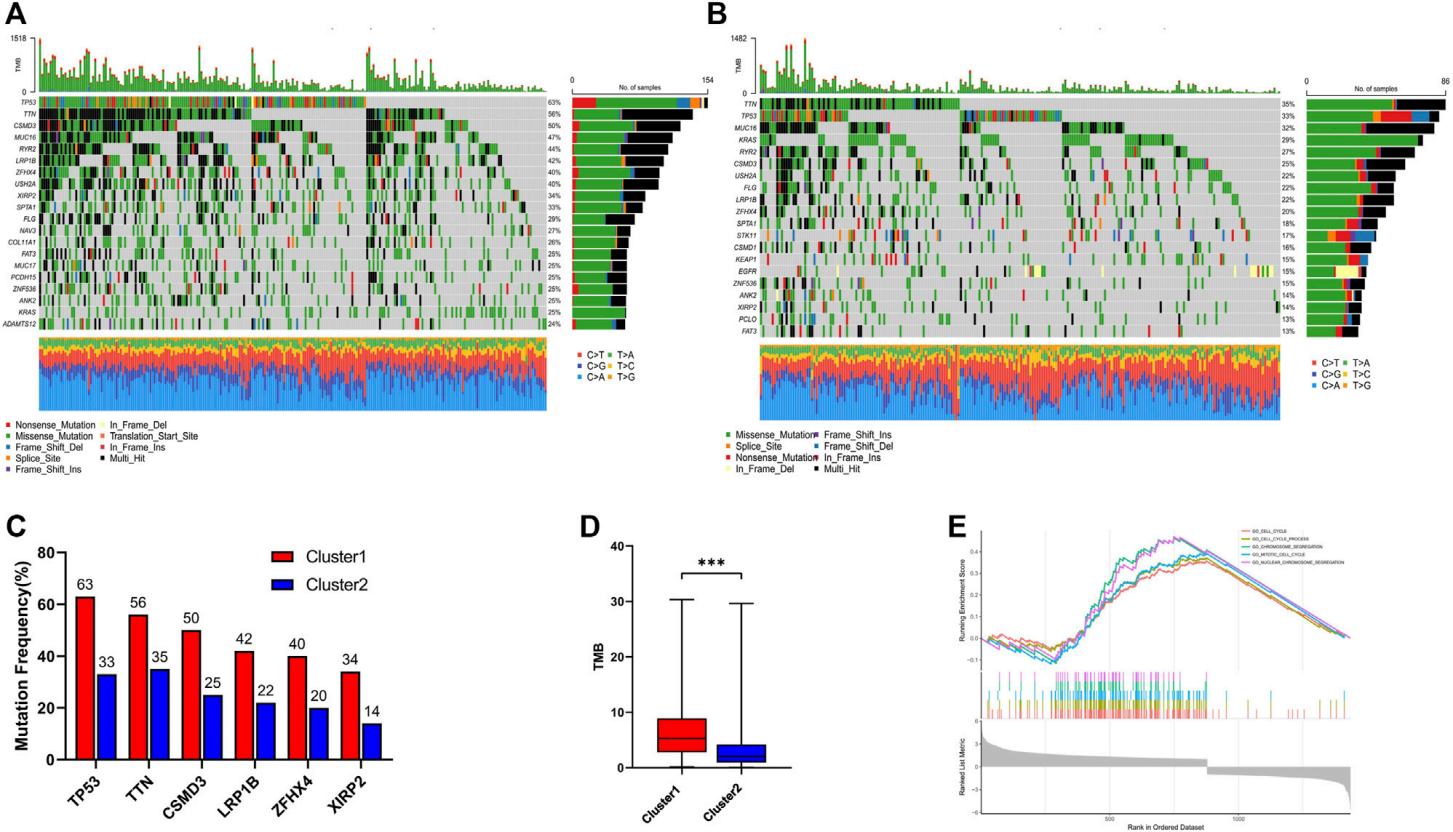
Genomic alterations and Gene set enrichment analysis between cluster 1 and cluster 2. **(A)** Landscape of mutation profiles in cluster 1; **(B)** Landscape of mutation profiles in cluster 2; **(C)** The six genes with the greatest variation in mutation frequency between cluster 1 and cluster 2; **(D)** The difference of tumor mutation burden between cluster 1 and cluster 2; **(E)** Top five most significant altered KEGG pathways in cluster 1 compared with cluster 2. *** means *p* < 0.001.

To further investigate the genetic landscape differences between the two subtypes, somatic mutation data in LUAD patients were used. In cluster 1, *TP53* was the most commonly mutated gene—with a frequency of 63%—followed by *TTN, CSMD3, MUC16,* and *RYR2* (Figure 4A). In cluster 2, the top five mutated genes with a relatively low mutation rate were *TTN, TP53, MUC16, KRAS,* and *RYR2* (Figure 4B). Although *TP53* was one of the most frequently mutated genes in both groups, the mutation rate was significantly different between cluster 1 and cluster 2 (63% vs. 33%; Figure 4C). In addition, different mutation frequencies of the same gene between the two clusters were also observed for *TTN, CSMD3, LRP1B, ZFHX4,* and *XIRP2* (Figure 4C), and the tumor mutation burden (TMB) of cluster 1 was significantly higher than in cluster 2 (Figure 4D). GSEA analysis was used to investigate the transcriptomic alterations between these two groups. The most prominent gene ontology terms in cluster 1 were cell cycle, cell cycle procession, chromosome segregation, mitotic cell cycle, and nuclear chromosome segregation (Figure 4E).

### Consensus clustering-based immune infiltrate analysis

**FIGURE 5:**
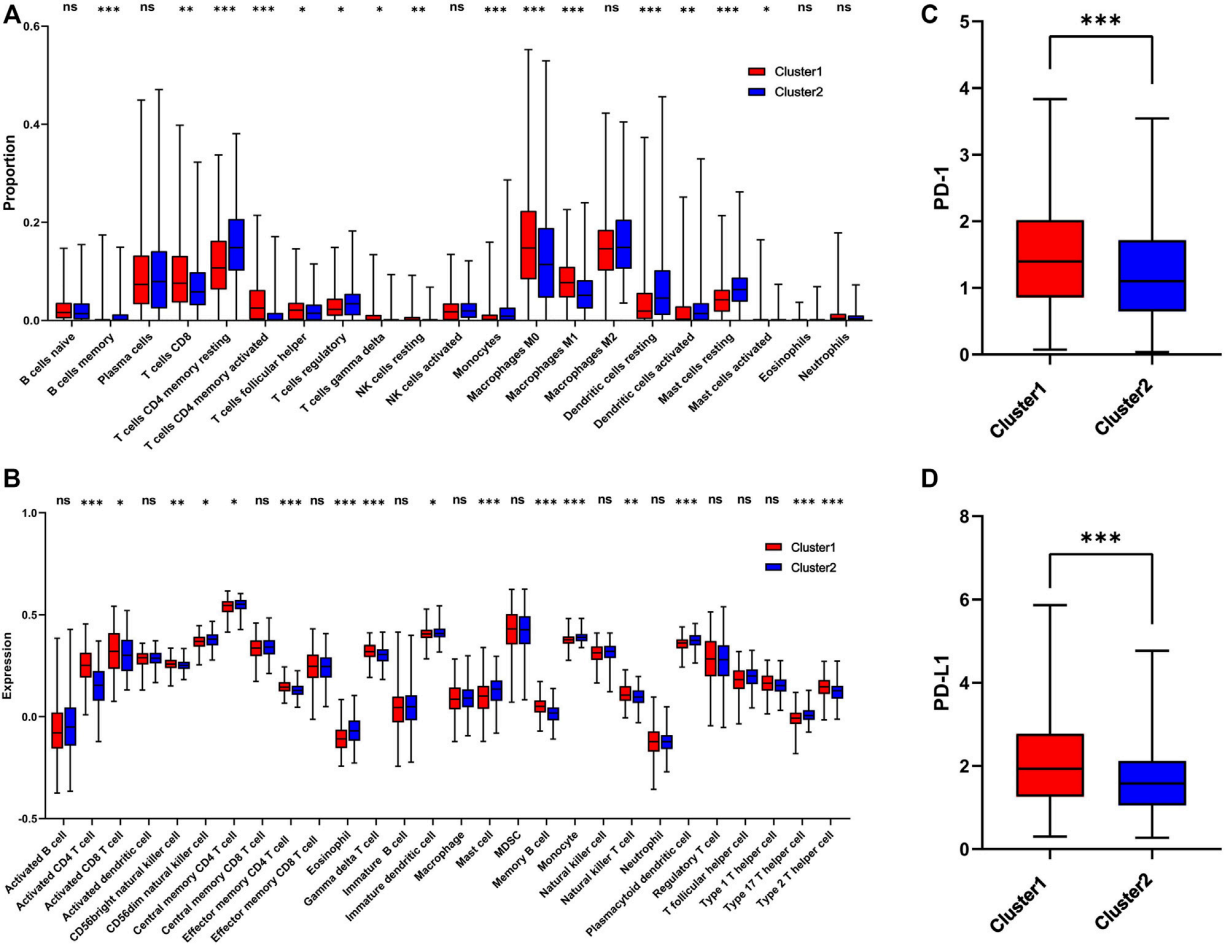
Immune profile alterations between cluster 1 and cluster 2. **(A)** The difference of 22 immune cell types between cluster 1 and cluster 2; **(B)** The difference of 28 immune cell types between cluster 1 and cluster 2; **(C)** The difference of PD-1 expression level between the cluster 1 and cluster 2; **(D)** The difference of PD-L1 expression level between cluster 1 and cluster 2. * means *p* < 0.05; ** means *p* < 0.01; *** means *p* < 0.001; ns means no significant difference.

The infiltration level of immune cells in the TME has been confirmed to play an important role in tumor progression and immunotherapy. To evaluate the difference in immune cell infiltration between the two subgroups, CIBERSORT and ssGSEA were performed in TCGA cohort. The CIBERSORT analysis showed that CD8+ T cells, activated CD4 T cells, M0 macrophages, and M1 macrophages were significantly upregulated in cluster 1, while memory B cells, CD4 memory resting T cells, regulatory T cells, and monocytes were downregulated (Figure 5A). The ssGSEA analysis revealed that activated CD4 T cells, activated CD8 T cells, NK cells, effector memory CD4 T cells, memory B cells, natural killer T cells, and Type 2 T helper cells were significantly upregulated, and Type 17 T helper cells were significantly downregulated, in cluster 1 (Figure 5B). Moreover, the expression of PD-1 and PD-L1 was also upregulated in cluster 1 (Figures 5C,D). The correlation of immune cells with 1C metabolism related-genes was also evaluated, and we found that the infiltration level of CD4 T cells was positively related to those genes (Supplementary Figures S5A,B). However, there was no difference was observed between the two clusters on the immune score (Supplementary Figures 6A–D). CIBERSORT and ssGSEA were also performed in GEO cohort. It showed that the infiltration level of immune cells in cluster 1 was higher than in cluster 2 (Supplementary Figures S7A,B).

### Correlation analysis of methylation enzymes with one-carbon metabolism-associated genes

**FIGURE 6:**
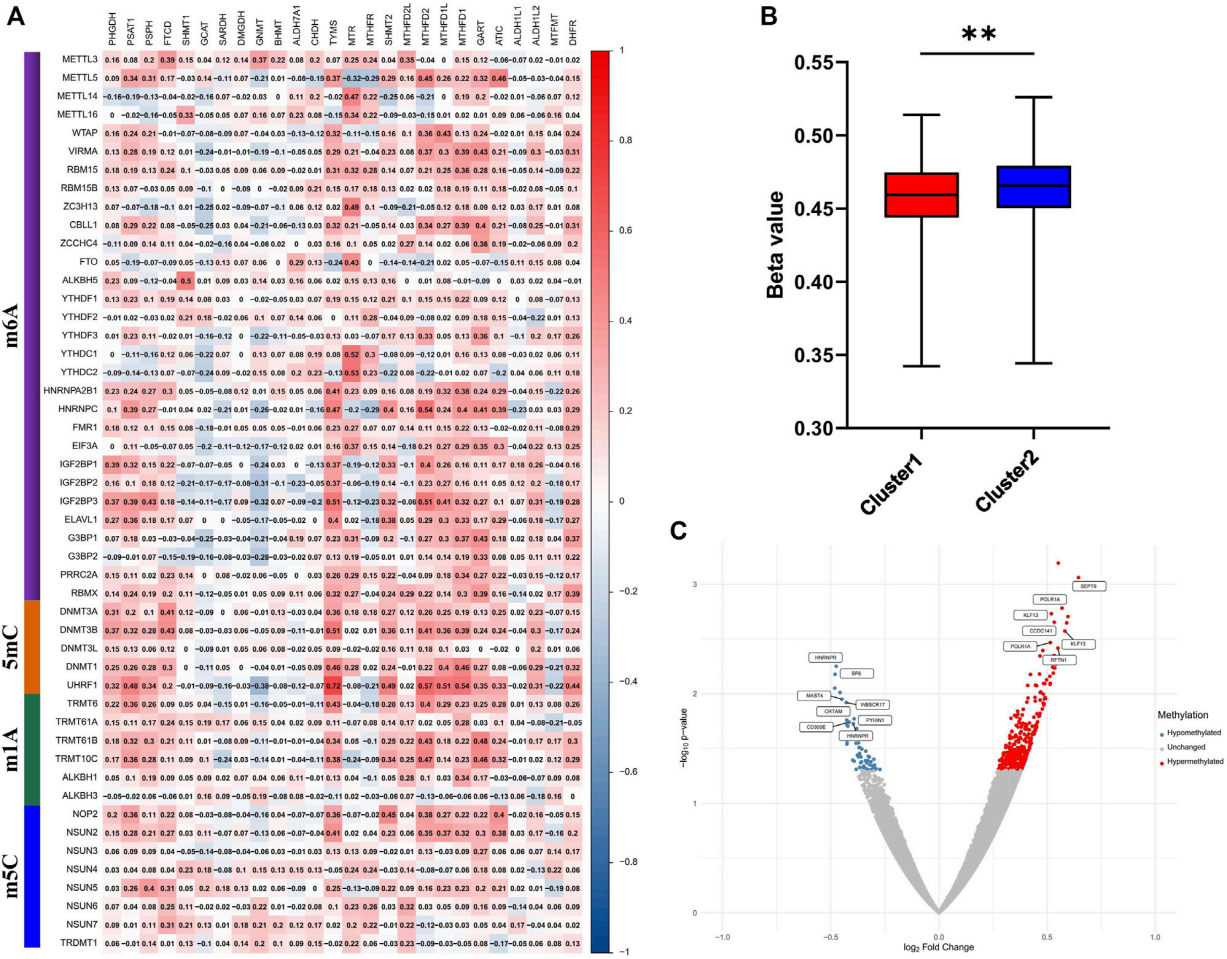
Correlation of one-carbon metabolism associated genes with methylation. **(A)** Correlation of 26 one-carbon metabolism associated genes with 49 methylation enzymes; **(B)** The difference of methylation level between cluster 1 and cluster 2; **(C)** Differential analysis of methylation sites between cluster 1 and cluster 2. ** means *p* < 0.01.

1C metabolism supports the biosynthesis and methylation of DNA and RNA by transferring 1C units. To explore the involvement of methylation with 1C metabolism-associated genes, 49 methylation enzymes were selected from previous studies (Zhang and Jia, 2018; Bohnsack et al., 2019; Chen and Zhang, 2020; Zhang C. et al., 2021). In addition, we further evaluated the correlation of methylation enzymes with 1C metabolism-associated genes. The results revealed that the expression of methylation enzymes was significantly positively associated with 1C metabolism-associated genes, such as *TYMS, MTR, MTHFR, SHMT2, MTHFD2L, MTHFD2, MTHFD1L, MTHFD1, GART, ATIC, PSAT1, PSPH, DHFR,* and *FTCD* (Figure 6A). We also investigated the difference in DNA methylation levels between the two groups and observed a significant downregulation of DNA methylation in cluster 1 (Figure 6B). In addition, a further differential analysis revealed that hypermethylation of *SEPT9* and *KLF13* was found in cluster 1, and hypomethylation of *HNRNPR* was also observed (Figure 6C).

### One-carbon metabolism-associated gene-based drug sensitivity analysis

**FIGURE 7:**
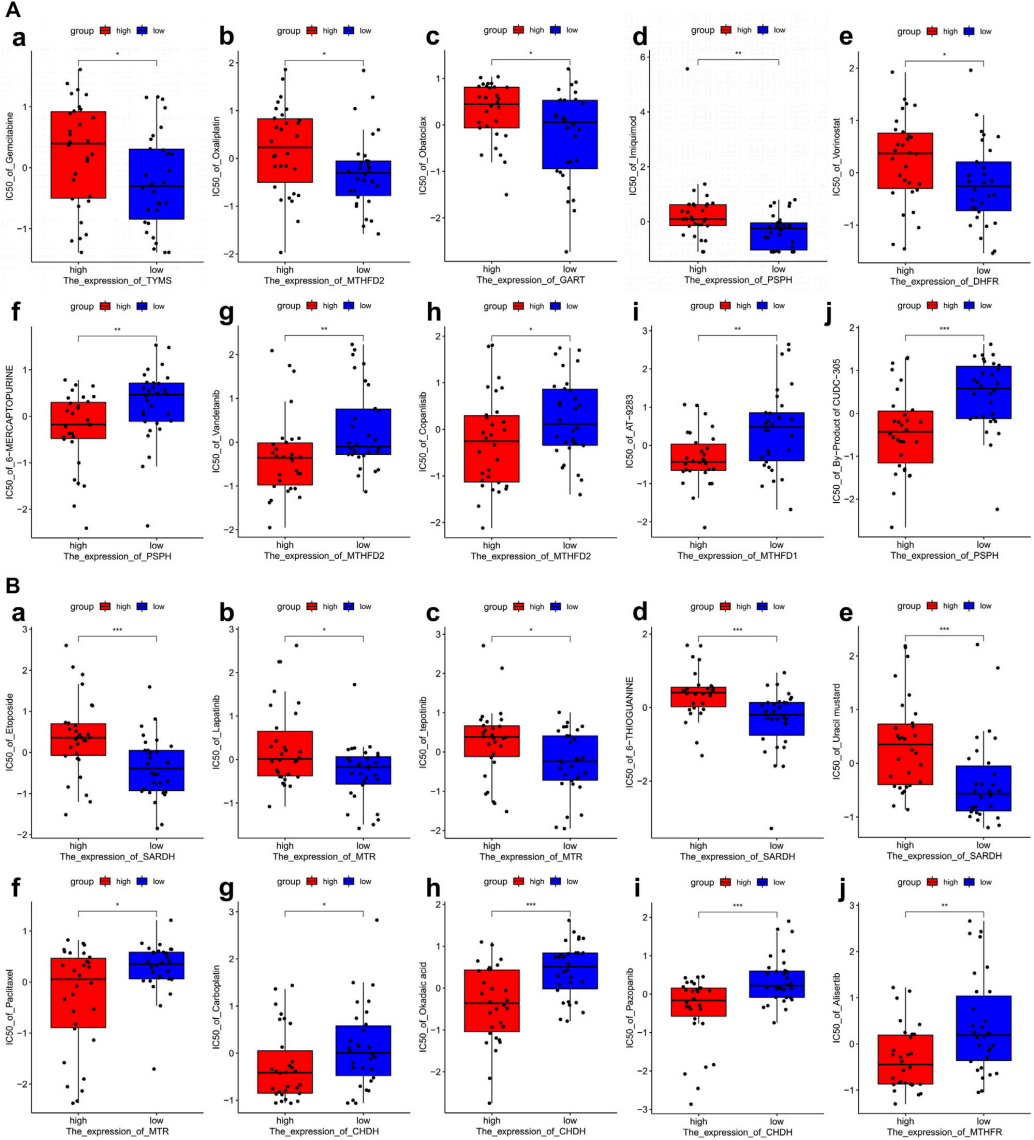
Drug sensitivity analysis of one-carbon metabolism associated genes. **(A)** Drug sensitivity analysis based on Cluster 1 expression pattern. (a–e) Five drugs with negatively related sensitivity, (f–j) Five drugs with positively related sensitivity; **(B)** Drug sensitivity analysis based on Cluster 2 expression pattern. (a–e) Five drugs with negatively related sensitivity, (f–j) Five drugs with positively related sensitivity.

To investigate the potential correlation between 1C metabolism-associated genes and drug sensitivity in multiple human tumor cell lines, a correlation analysis was performed in the CellMiner™ database. Cells with the expression pattern of cluster 1 were negatively associated with drug sensitivity to gemcitabine, oxaliplatin, obatoclax, imiquimod, and vorinostat (Figures 7Aa–e), and positively correlated to drug sensitivity to 6-mercaptopurine, vandetanib, copanlisib, AT-9283 and byproducts of CUDC-305 (Figures 7Af–g). Cells with the expression pattern of cluster 2 were negatively correlated with drug sensitivity to etoposide, lapatinib, tepotinib, 6-thioguanine, and uracil mustard (Figures 7Ba–e), but were positively correlated with drug sensitivity to paclitaxel, carboplatin, okadaic acid, pazopanib and alisertib (Figures 7Aa–e). Patients in cluster 1 were also insensitive to paclitaxel and carboplatin, suggesting that patients in cluster 1 are likely resistant to gemcitabine, paclitaxel, oxaliplatin, and carboplatin treatment.

### One-carbon metabolism-associated genes are positively correlated with immunotherapy sensitivity

**FIGURE 8:**
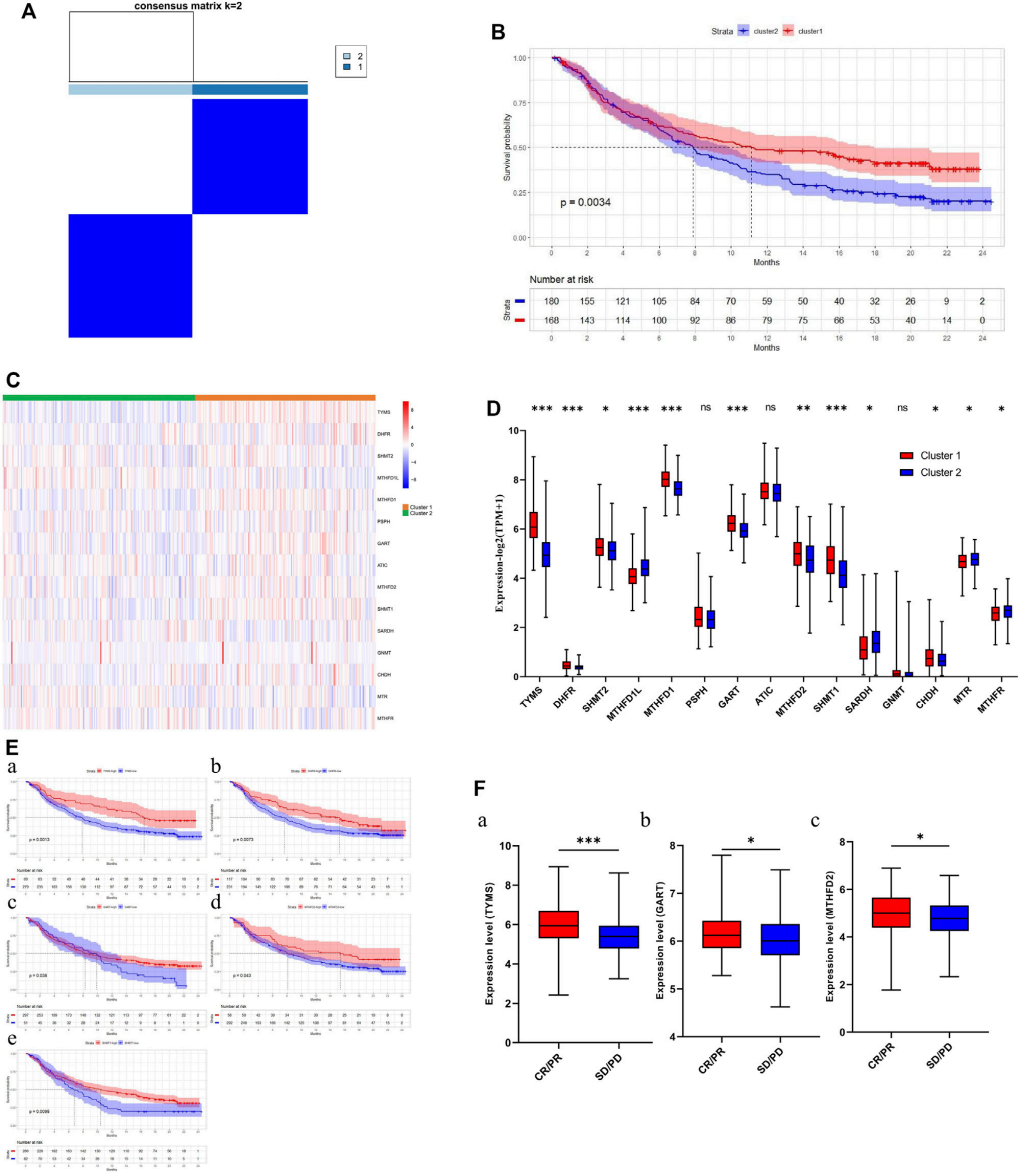
Association of one-carbon metabolism related genes with immunotherapy. **(A)** The consensus matrix shows patients with two distinct one-carbon metabolism statuses; **(B)** Kaplan-Meier curves for overall survival in two clusters (Log-rank test); **(C)** Heatmap of one-carbon metabolism associated genes expression level in two clusters; **(D)** The expression difference of one-carbon metabolism associated genes in two clusters; **(E)** Kaplan-Meier survival curves of five genes associated with superior OS; **(F)** The expression difference of three one-carbon metabolism associated genes between CR/PR group and SD/PD group. * means *p* < 0.05; ** means *p* < 0.01; *** means *p* < 0.001; ns means no significant difference.

According to the results above, cluster 1 in the LUAD cohort is resistant to chemotherapy but may be sensitive to immunotherapy. We therefore explored the relationship between 1C metabolism-associated genes and immunotherapy in the IMvigor210 cohort. Consensus clustering was also performed, and two clusters (cluster 1 and cluster 2) were identified among patients in the IMvigor210 cohort (Figure 8A). A Kaplan-Meier analysis showed that for patients treated with immunotherapy, cluster 1 had a superior OS compared with cluster 2 (median OS: 11.2 vs. 7.8 months, *p* = 0.0034; Figure 8B). The expression pattern of cluster 1 in the IMvigor210 cohort was similar to that of cluster 1 in the LUAD cohort (Figures 8C,D). A Kaplan-Meier analysis revealed that high expression of *DHFR, TYMS, GART, MTHFD2* and *SHMT1* were correlated with a superior OS (Figure 8E). In addition, the expression levels of *TYMS, GART,* and *MTHFD2* in patients with a complete or partial response were higher than for patients with stable or progressive disease (Figure 8F). In addition, ROC curves were drawn to assess the specificity and sensitivity of 1C metabolism related-genes with the value of 0.69 (Supplementary Figure S2C).

## Discussion

1C metabolism has been shown to play a role in the occurrence, development, and treatment of multiple cancers. Many 1C metabolic enzymes have been observed upregulated in cancer tissues compared with adjacent normal tissues, and are closely associated with cancer prognosis. However, the literature on 1C metabolism in LUAD patients is sparse. In the present study, we evaluated the expression levels of 1C metabolism-related genes and the correlation with LUAD prognosis. Unsupervised clustering analysis was performed to classify the samples into cluster 1 and cluster 2. We found that cluster 1 was characterized by inferior OS, increased TMB, high PD-1 and PD-L1 expression, as well as enhanced immune infiltration. In addition, 1C metabolism-related genes were positively correlated with the expression of methylation enzymes, and lower methylation levels were observed in cluster 1. Patients in cluster 1 were also resistant to chemotherapy drugs, including pemetrexed, gemcitabine, paclitaxel, etoposide, oxaliplatin, and carboplatin. We also found that 1C metabolism-related genes were positively correlated with immunotherapy sensitivity.

In our study, 1C metabolism-related genes were selected according to previous studies. The expression levels of these genes were evaluated, and twenty genes were upregulated in tumor tissues, while two genes were downregulated. The upregulated genes included *PSAT1, PSPH, FTCD, SHMT1, SHMT2, MTHFD2L, MTHFD2, MTHFD1L, MTHFD1, GCAT, DMGDH, ALDH7A1, CHDH, TYMS, GART, ATIC, ALDH1L1, ALDH1L2, DHFR,* and *MTFMT*, while the downregulated genes included *GNMT* and *MTHFR*. A Kaplan-Meier analysis revealed that 17 genes were associated with prognosis. Among these genes, nine genes were identified as risk factors while the other eight genes were considered protective factors. A univariate Cox regression analysis identified six risk-associated genes and four protective genes. Consensus clustering was performed, and 497 LUAD patients were classified into two clusters. We found that, compared with cluster 2, cluster 1 exhibited the opposite expression pattern and a worse OS.

The genetic landscape of these two groups was also investigated. We observed that somatic mutations were more frequent in the high-risk group. The mutation rates of *TP53, TTN, CSMD3, LRP1B, ZFHX4* and *XIRP2* were significantly higher in the high-risk group compared with the low-risk group. In addition, a heavier TMB was also observed in the high-risk group. Furthermore, the GSEA results suggested that the pathways, which were associated with cell cycle and chromosome segregation, were significantly enriched in the high-risk group. *TP53* (p53) is one of the most common tumor suppressor genes in human cancers. The p53 protein plays an antitumor role by repairing DNA damage, regulating metabolism, normalizing reactive oxygen species levels, modulating expression of non-coding RNAs, and promoting autophagy or ferroptosis (Duffy et al., 2017). TP53 mutations were also positively correlated with PD-L1 expression, TMB, and clinical benefit of PD-1 inhibitors (Dong et al., 2017). In addition, mutant *TTN, CSMD3, LRP1B* was also positively correlated with response rate to immunotherapy (Jia et al., 2019; Brown et al., 2021; Lu et al., 2021). Therefore, LUAD patients in cluster 1 may benefit from immunotherapy treatment.

To investigate the difference in the TME between these two groups, CIBERSORT and ssGSEA were performed. The results revealed that CD8^+^ T cells, CD4+ T cells, NK cells, Type 2 T helper cells and M1 macrophages were significantly upregulated in the high-risk group, while regulatory T cells were downregulated. Furthermore, the expression of PD-1 and PD-L1 was significantly upregulated. The TME is closely related to the occurrence and progression of tumors, and influences immunotherapy efficacy (Dai et al., 2021). A previous study suggests that CD8+ T cells, CD4+ T cells, NK cells and M1 macrophages influence the clinical benefit of immunotherapy, while Treg cells impair the immunotherapy efficacy (Petitprez et al., 2020). In addition, PD-1 and PD-L1 have also been considered as protective biomarkers for immunotherapy (Petitprez et al., 2020). Previous studies suggested that 1C metabolism was associated the development of immune system (Ducker and Rabinowitz, 2017). The activation of immune cells, especially T cells, required an ample supply of 1C units (Ron-Harel et al., 2016). Therefore, we speculated that 1C metabolism related genes may contribute to the accumulation of folate in TME, which may support the development and activation of immune cells. On the other way, it also may be an underlying competitive absorption of 1C units between the tumor cells and immune cells. Based on these results, we speculate that patients in cluster 1 may benefit from immunotherapy.

1C metabolism generates 1C units to support methylation reactions. To investigate the relationship between 1C metabolism and DNA and RNA methylation, we calculated the correlation of enzymes in the 1C metabolism pathway with 49 methylation enzymes, and the results suggested that 1C metabolism genes are generally positively correlated with methylation enzymes, such as “writers”, “readers”, and “erasers”. A previous review suggests that RNA modifications, including m6A, m1A and m5C, plays an important role in the occurrence and development of lung cancer (Teng et al., 2021). Considering the function of DNA methylation enzymes, we further evaluated the DNA methylation levels between two subgroups and found low methylation levels in the high-risk group. DNA hypomethylation promotes the development of cancer partly by activating oncogenic potential genes (Van Tongelen et al., 2017). A differential analysis suggests that hypermethylation occurs in *SEPT9* and *KLF13*, while hypomethylation occurs in *HNRNPR*. *SEPT9* and *KLF13* have been shown to be antitumor genes in previous studies (Jiao et al., 2019; Yao et al., 2020). The hypermethylation of these genes impairs expression and promotes tumor development. *HNRNPR* contributes to the proliferation and metastasis of gastric cancer (Chen et al., 2019), whereby *HNRNPR* hypomethylation leads to tumor proliferation and metastasis. According to these findings, we speculated that 1C metabolism may play an important role in both methylation and demethylation. On the one hand, the high expression level of 1C metabolism related genes accelerate the generation of 1C units in tumor cells. The abundant methyl groups provide the needs of the methylation of DNA, RNA and proteins, which have been proved by numerous studies (Robertson and Jones, 2000; Van Tongelen et al., 2017; Chen et al., 2019; Jiao et al., 2019; Yao et al., 2020; Dai et al., 2021; Song P. et al., 2021). On the other hand, high expressing 1C metabolism related genes can absorb redundant methyl groups generated by demethylation, thereby promoting the demethylation process. Thus, we speculated that 1C metabolism related genes may contribute to the redistribution of 1C units, by which the important biological processes are influenced. In addition, previous studies indicate that patient tumors with low levels of DNA methylation and high expression of RNA methyltransferases respond better to immunotherapy (Emran et al., 2019; Zhang et al., 2021b). We therefore hypothesized that patients in cluster 1 may benefit from immunotherapy.

We further explored the potential correlation between 1C-related gene expression and drug sensitivity in the CellMiner database. Based on the expression pattern of cluster 1, tumor cells exhibited lower sensitivity to gemcitabine and oxaliplatin, but a higher sensitivity to 6-mercaptopurine. However, tumors with a cluster 2 expression pattern were sensitive to paclitaxel and carboplatin, while resistant to etoposide and lapatinib. Gemcitabine, paclitaxel, etoposide, oxaliplatin, and carboplatin are common drugs for the treatment of NSCLC, however, only a subset of patients benefit from these drugs (Hu et al., 2016; Cui et al., 2020; Esim et al., 2020; Zhang et al., 2021a). Lapatinib is a dual tyrosine kinase inhibitor that has been shown to have promising antitumor effects in NSCLC (Huijberts et al., 2020). 6-mercaptopurine is also an antitumor drug, and the achievement of its therapeutic activity requires the enzymatic conversion to thio-GMP to displace thio-GTP in RNA and DNA (Karran and Attard, 2008). Pemetrexed plays an important role in the treatment of LUAD and has a response rate of 30% (Postmus, 2002). Pemetrexed’s antitumor function is achieved by inhibiting three key enzymes in the 1C metabolism pathway: thymidylate synthase (TYMS), dihydrofolate reductase (DHFR), and glycinamide ribonucleotide formyltransferase (GART). A recent study indicates that *MTHFD2* overexpression is involved in resistance to pemetrexed (Yao et al., 2021). We found these four genes to be significantly upregulated in tumor tissues, especially in cluster 1, and were associated with a poor prognosis. Therefore, we can reasonably deduce that LUAD patients with high expression levels of 1C metabolism-related genes may be inherently insensitive to pemetrexed treatment. Likely as a result of the opposite expression pattern of 1C metabolism-related genes in these two groups, the opposite drug sensitivity pattern existed among these two clusters. Patients in cluster 1 were resistant to chemotherapeutic drugs, including pemetrexed, gemcitabine, paclitaxel, oxaliplatin, and carboplatin. Thus, patients in cluster 1 may benefit from immunotherapy.

We speculated that patients in cluster 1 could benefit from immunotherapy. We therefore investigated the relationship between 1C metabolism-related genes and immunotherapy in the IMvigor210 cohort. *DHFR, TYMS, GART, MTHFD2* and *SHMT1* were correlated with a superior OS to immunotherapy. The expression levels of *TYMS, GART,* and *MTHFD2* were also higher in patients with a complete or partial response compared with patients who had stable or progressive disease. Thus, we speculated that 1C metabolism-related genes may play a role in immunotherapy response and lead to a clinical benefit from immunotherapy.

Several limitations exist in our study. Although the results were substantiated in both TCGA and the IMvigor210 cohort, they were not confirmed in LUAD patients who were treated with immunotherapy because of the insufficient transcriptome data from clinical trials. Consequently, different data sets were used in this study. To reduce bias, external validation in larger cohorts is required to validate these findings. Lastly, *in vivo* and *in vitro* experiments are needed to explore the potential mechanisms.

Taken together, our study demonstrates that 1C metabolism-related genes possess potential as therapeutic targets as well as biomarkers of prognosis of immunotherapy in LUAD. Based on the expression pattern of 1C metabolism-related genes, LUAD patients can be classified into two subtypes. Specific subtype characteristics provide information for LUAD clinical management and decision-making. Our findings provide new insight into the mechanisms associated with poor LUAD prognosis, predict efficacy of several therapeutic drug, as well as assist in identifying biomarkers for immunotherapy in LUAD patients.

## Data Availability

The original contributions presented in the study are included in the article/Supplementary Material, further inquiries can be directed to the corresponding authors.

## Supplementary material

SUPPLEMENTARY FIGURE S1 Kaplan-Meier survival curves and Univariate Cox regression analysis of one-carbon metabolism associated genes. **(A)** Kaplan-Meier survival curves of 13 genes associated with inferior OS; **(B)** Kaplan-Meier survival curves of 10 genes associated with superior OS; **(C)** Kaplan-Meier survival curves of 3 genes not associated with OS; **(D)** Univariate Cox regression analysis of 26 one-carbon metabolism associated genes.

SUPPLEMENTARY FIGURE S2 ROC curves of 1C metabolism related-genes in TCGA, GEO, and IMvigor210 cohort. **(A)** The 1-year, 5-year, and 10-year ROC curve in TCGA cohort; **(B)** The 1-year, 5-year, and 10-year ROC curve in GEO cohort; **(C)** The ROC curve in IMvigor210 cohort.

SUPPLEMENTARY FIGURE S3 Lasso regression and the establishment of risk score model. **(A,B)** Lasso regression for 1C metabolism related-genes in LUAD patients; **(C,D)** The establishment of risk score model in LUAD patients; **(E)** The expression difference of one-carbon metabolism associated genes in high- and low-risk groups; **(F)** Kaplan-Meier curves for overall survival in two subgroups (Log-rank test). * means *p* < 0.05; ** means *p* < 0.01; *** means *p* < 0.001; ns means no significant difference.

SUPPLEMENTARY FIGURE S4 Consensus clustering for one-carbon metabolism associated genes in LUAD patients. **(A)** The consensus matrix shows patients with two distinct one-carbon metabolism statuses; **(B)** Heatmap of one-carbon metabolism associated genes expression level in two clusters; **(C)** The expression difference of one-carbon metabolism associated genes in two clusters; **(D)** Kaplan-Meier curves for overall survival in two clusters (Log-rank test). * means *p* < 0.05; ** means *p* < 0.01; *** means *p* < 0.001; ns means no significant difference.

SUPPLEMENTARY FIGURE S5 Correlation of one-carbon metabolism associated genes with immune cells. **(A)** Correlation of 26 one-carbon metabolism associated genes with 22 immune cells; **(B)** Correlation of 26 one-carbon metabolism associated genes with 28 immune cells.

SUPPLEMENTARY FIGURE S6 ESTIMATE analysis between cluster 1 and cluster 2. **(A)** The ESTIMATE score between cluster 1 and cluster 2; **(B)** The Tumor purity between cluster 1 and cluster 2; **(C)** The immune score between cluster 1 and cluster 2; **(D)** The stromal score between cluster 1 and cluster 2. ns means no significant difference.

SUPPLEMENTARY FIGURE S7 Immune profile alterations between cluster 1 and cluster 2. **(A)** The difference of 22 immune cell types between cluster 1 and cluster 2; **(B)** The difference of 28 immune cell types between cluster 1 and cluster 2. * means *p* < 0.05; ** means *p* < 0.01; *** means *p* < 0.001; ns means no significant difference.
