# Supplementary figures and images for: Comprehensive analyses of one-carbon metabolism related genes and their association with prognosis, tumor microenvironment, chemotherapy resistance and immunotherapy in lung adenocarcinoma
Source: Front Mol Biosci. 2022 Nov 11;9:1034208. doi: 10.3389/fmolb.2022.1034208 (PMC9699278; doi:10.3389/fmolb.2022.1034208)

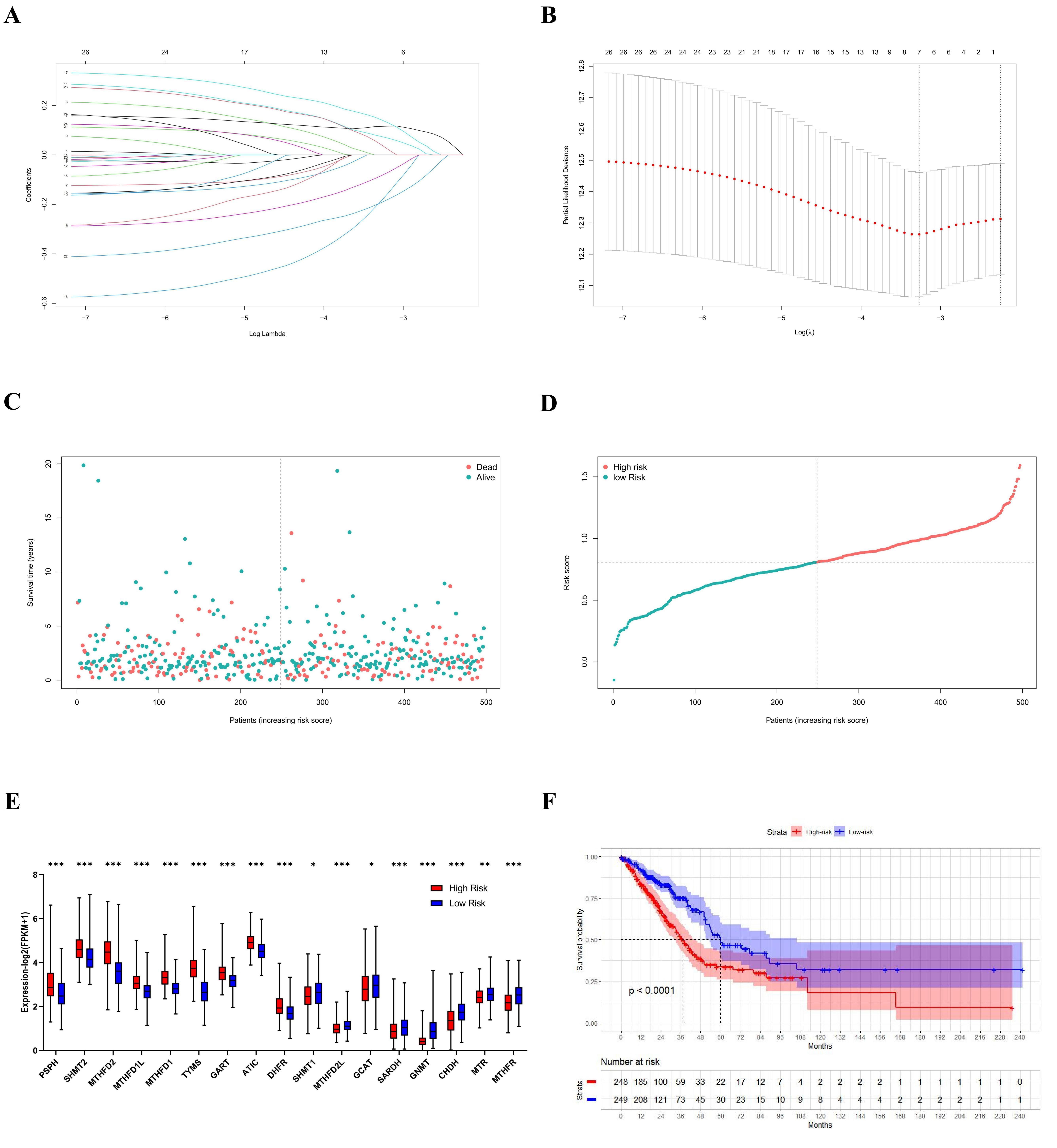

Supplement: Supplementary file 1 [file Image3.JPEG]

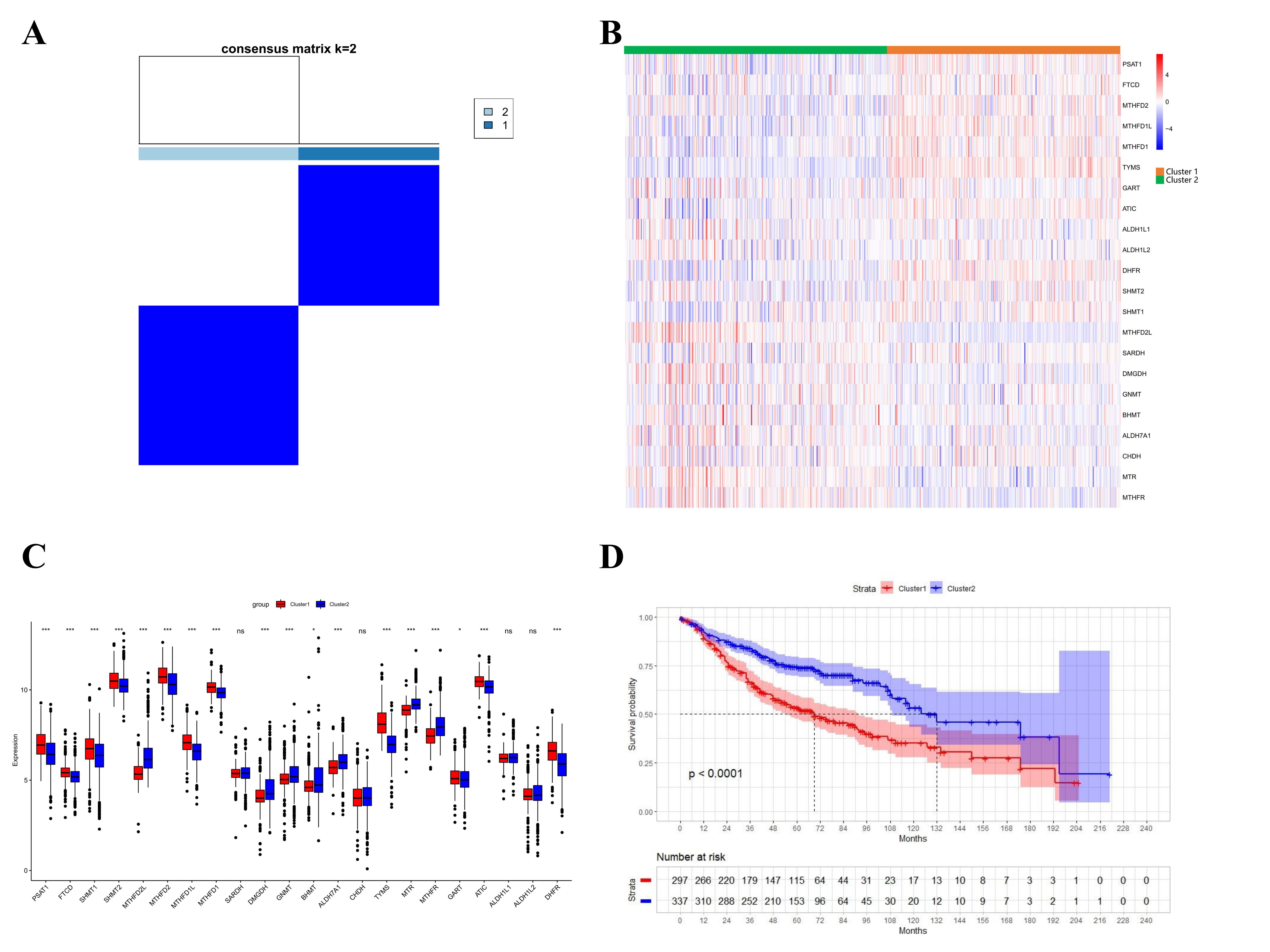

Supplement: Supplementary file 3 [file Image4.JPEG]

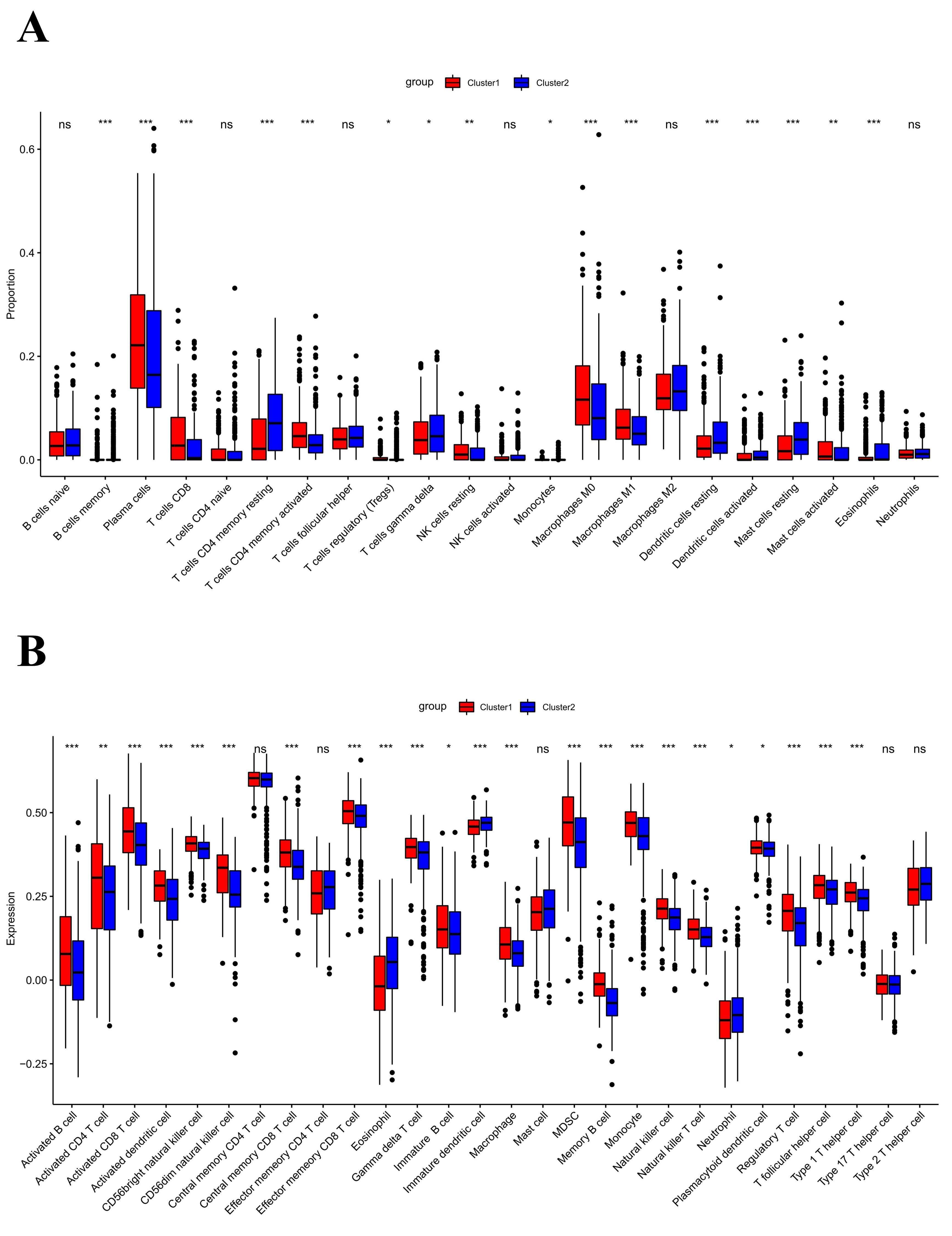

Supplement: Supplementary file 4 [file Image7.JPEG]

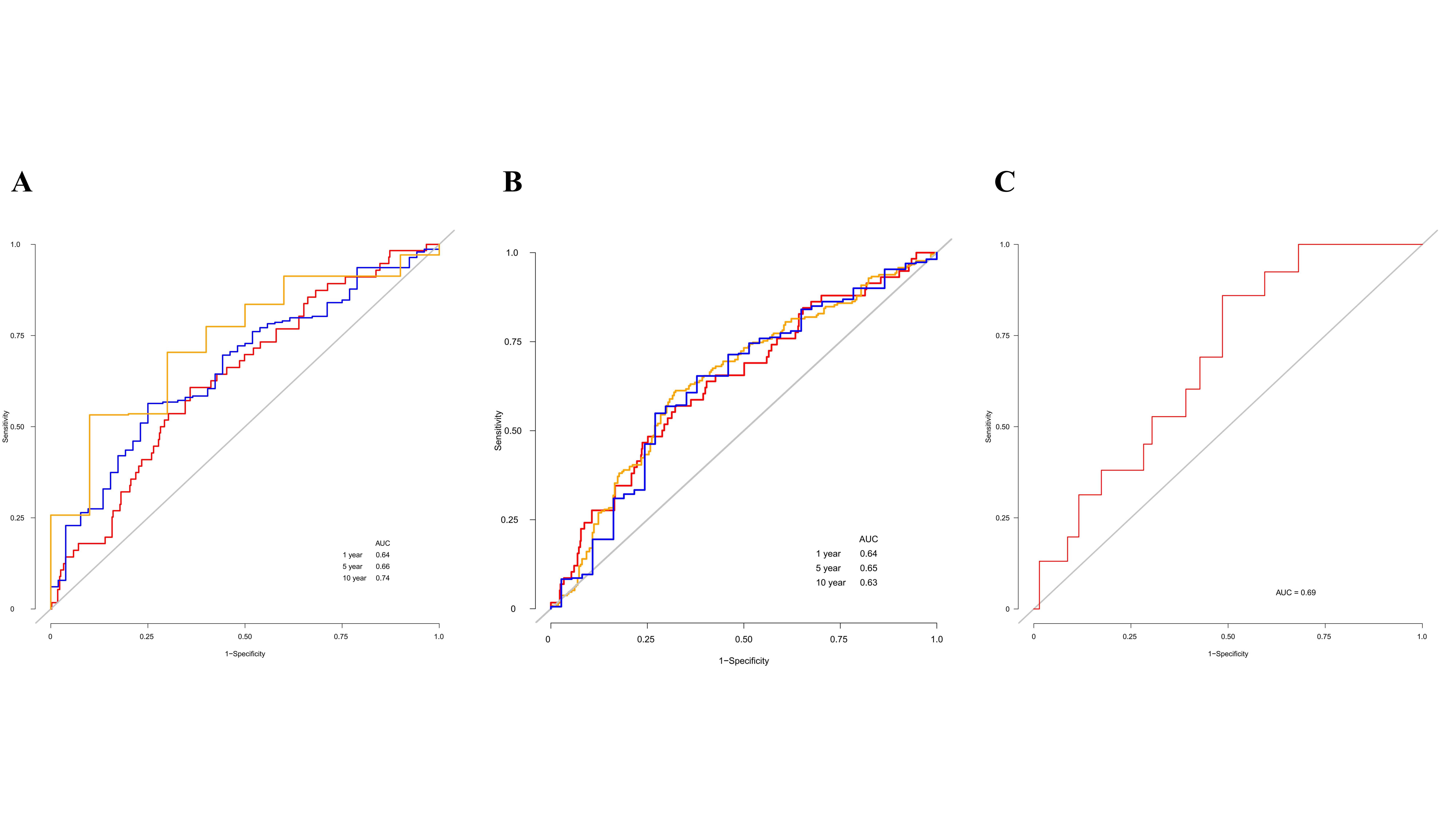

Supplement: Supplementary file 5 [file Image2.JPEG]

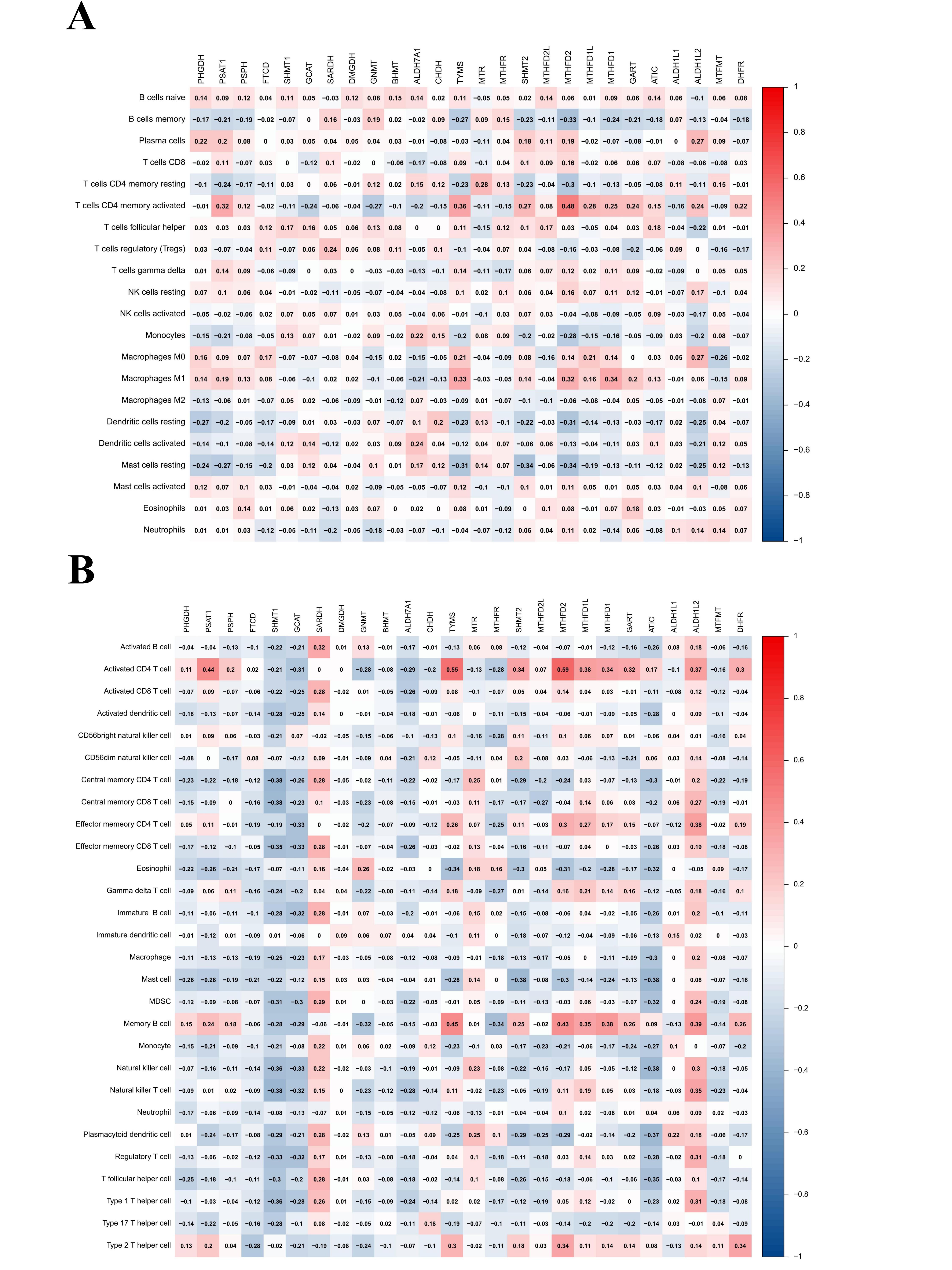

Supplement: Supplementary file 6 [file Image5.JPEG]

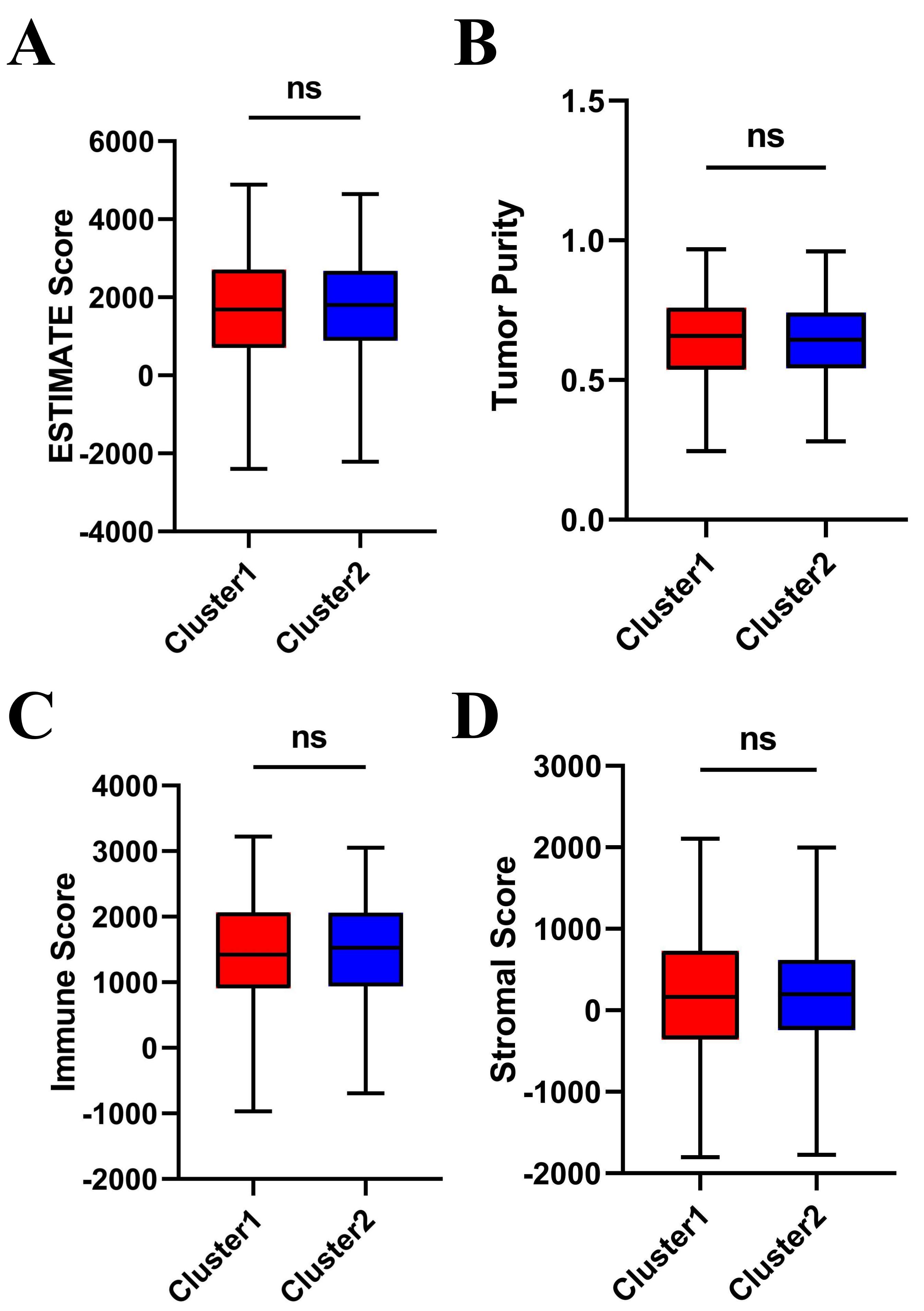

Supplement: Supplementary file 7 [file Image6.JPEG]
